# Cytotoxic Effect of Icaritin and Its Mechanisms in Inducing Apoptosis in Human Burkitt Lymphoma Cell Line

**DOI:** 10.1155/2014/391512

**Published:** 2014-05-07

**Authors:** Zi-Jian Li, Can Yao, Su-Fang Liu, Long Chen, Ya-Ming Xi, Wen Zhang, Guang-Sen Zhang

**Affiliations:** ^1^Division of Hematology, The First Affiliated Hospital, Lanzhou University, Lanzhou, Gansu 730000, China; ^2^Division of Nephrology, The First Affiliated Hospital, Lanzhou University, Lanzhou, Gansu 730000, China; ^3^Division of Hematology, The Second Xiang-Ya Hospital, Central South University, Changsha, Hunan 410011, China

## Abstract

Icaritin (ICT), a hydrolytic product of icariin from *Epimedium genus*, exhibits antitumor activities in several human solid-tumor and myeloid leukemia cells with extensive influence on various cell signal molecules, such as MAPKs being involved in cell proliferation and Bcl-2 participating in cell apoptosis. However, the effect of icaritin on Burkitt Lymphoma has not been elucidated. In the present study, we first screened the potential effect of icaritin on Burkitt lymphoma Raji and P3HR-1 cell lines and found that icaritin showed cytotoxicity in both cell lines. We further found that icaritin could significantly inhibit Raji cells proliferation with S-phase arrest of cell cycle and induced cell apoptosis accompanied by activation of caspase-8 and caspase-9 and cleavage of PARP. We also observed that icaritin was able to decrease Bcl-2 levels, thus shifting the Bcl-2/Bax ratio, and it could obviously reduce c-Myc, a specific molecular target in Burkitt lymphoma. Our findings demonstrated that icaritin showed cytotoxicity, inhibited cell growth, caused S arrest, and induced apoptosis in Burkitt lymphoma cells and provided a rationale for the further evaluation of icaritin for Burkitt lymphoma therapy.

## 1. Introduction

Burkitt lymphoma (BL) is a highly aggressive B-cell lymphoma with extremely short doubling time that presents usually in extranodal sites or as an acute leukemia. As sporadic BL, the incidence is 1-2% of all Non-Hodgkin's lymphoma (NHL) and accounts for 30–50% of all childhood lymphoma in Western countries [1]. To the current knowledge, Burkitt lymphoma is a so-called double hit (DH) lymphoma that is defined by a chromosomal breakpoint affecting the* MYC*/8q24 locus in combination with another recurrent breakpoint, mainly a t(14;18)(q32;q21) involving* BCL2*. By the current standard regimens consists of cyclical intensive chemotherapy and aggressive intrathecal prophylaxis, Burkitt lymphoma have become curable in most patients. However, in high-risk relapsed patients with BL, the prognosis of BL is still very poor. Thus, further studies are needed to better define the therapy regime in older patients and in some relapsed or refectory cases and to elucidate novel anti-Burkitt lymphoma agents with fewer side effects. Icaritin is a hydrolytic product of icariin that is extracted from* Epimedium* brevicornum, which is a traditional Chinese medicine (TCM). Icaritin has showed significant antitumor activities with minor side effects in various neoplasms, such as inducing apoptosis in human endometrial cancer Hec1A cells [2], leading to cell growth inhibition, G1 arrest, and apoptosis of prostate cancer PC-3 cells [3], resulting in cell cycle arrest at the G2/M phase, apoptotic cell death [4] and enhancing radio sensitivity [5] in breast cancer cells, inhibiting growth [6], and reversing multidrug resistance [7] of hepatoma HepG2 cells. For hematopoietic neoplasms, Li and his team demonstrated the antitumor effect of icaritin in acute myeloid leukemia cells [8], and we reported that icaritin showed potent antileukemia activity on chronic myeloid leukemia* in vitro* and* in vivo* [9]. These results indicate that icaritin may possess broad-spectrum antitumor activities to various malignancies including hematopoietic malignancies. However, there are still no reports on the effect of icaritin on lymphoid neoplasms. This study was aimed to illustrate the cytotoxic effects of icaritin on human Burkitt lymphoma cell lines, for example, Raji and P3HR-1. We have further explored the changes of apoptosis related proteins such as caspase-9, casepase-8, PARP, and the two critical “double hit” factors that are c-Myc and Bcl-2. Herein, our study demonstrated that icaritin showed cytotoxicity, inhibited the two critical factors, c-Myc and Bcl-2, in Burkitt lymphoma cells, and provided a rational for the further preclinical and clinical evaluation of icaritin for Burkitt lymphoma therapy.

## 2. Materials and Methods

### 2.1. Chemicals and Reagents

Icaritin with a purity of up to 99.5% was a gift from Dr. Meng-kun at Shen-ao Gene Company (Beijing, China). Icaritin was dissolved in dimethyl sulfoxide (DMSO) and filtered through a 0.22 *μ*m filter (Millipore). A stock solution of 20 mM icaritin was kept at −20°C, and serial dilutions from 2 *μ*M to 8 *μ*M were freshly prepared in DMSO for each experiment. The final concentration of DMSO in the culture media was maintained at less than 0.1%, which had no significant effect on cell growth. The anti-Bcl-2 (sc-7382), anti-Bax (sc-7480), anti-c-Myc (sc-764), and anti-actin (sc-47778) antibodies were purchased from Santa Cruz Biotechnology (Santa Cruz, CA, USA). The anti-cleaved-PARP (no. 9541), anti-caspase-9 (no. 9502), and anti-caspase-8 (no. 9746) antibodies were purchased from Cell Signaling Technology (Boston, MA, USA).

### 2.2. Cell Lines

The two Burkitt lymphoma cell lines, Raji cells (ATCC: CCL-86) and P3HR-1 cells (ATCC: HTB-62), were cultured in RPMI 1640 (Hyclone) supplemented with 10% heat-inactivated newborn calf serum (NCS, Hyclone,), 50 IU/mL penicillin, and 50 mg/mL streptomycin in a humidified atmosphere of 5% CO_2_ at 37°C. All experiments were performed using logarithmically growing cells (4–6 × 10^5^cells/mL).

### 2.3. Cell Viability Assay

The* in vitro* toxicology assay was performed using the MTT based method. Briefly, cells (10^4^ cells/well) were seeded in 96-well microplates and were exposed to different concentrations of icaritin (0 *μ*M, 2 *μ*M, 4 *μ*M, and 8 *μ*M) for 24 h, 48 h, and 72 h, respectively. The MTT were added (0.5 mg/mL), and the cells were incubated for 4 h. After centrifugation for 10 min, the culture medium was discarded and formazan products were dissolved with 150 *μ*L of DMSO for 5 min with shaking at room temperature. The absorbance was measured at 492 nm using a microplate reader. Three reduplicate wells were used for each concentration point, and experiments were repeated three times. IC50 values were determined by plotting a linear regression curve. The present cell viability was calculated as follows:

(1) $$\begin{array}{c} \text{Cell}\;\text{viability}\left(\%\right)=\frac{\text{OD}\;\text{of}\;\text{treatment}}{\text{OD}\;\text{of}\;\text{control}}\times 100. \end{array}$$

### 2.4. Cell Cycle and Apoptosis Assay

Raji cells were seeded in a 6-well plate and treated with a range of concentrations of icaritin (0 *μ*M, 2 *μ*M, 4 *μ*M, 8 *μ*M) for 48 h. After treatment, the cells were collected, washed twice with prechilled PBS, and fixed in 75% cold ethanol overnight. Ethanol-fixed cells were washed with PBS and incubated with 100 *μ*g/mL of RNaseA at 37°C for 30 min followed by 50 *μ*g/mL of propidium iodide (PI) at room temperature for 30 min. Flow cytometry analysis was performed using a FACSCalibur flow cytometer (BD Biosciences, San Jose, CA, USA). Data were analyzed using the ModFit LT 3.0 software packages (Verity Software House, Topsham).

Cell apoptosis was detected with the Annexin V-FITC/PI Apoptosis Detection Kit (Keygen, Nanjing, China) according to the manufacturer's instructions. Data acquisition was performed using a FACSCalibur Flow cytometer and analyzed with CellQuest software.

### 2.5. Western Blot Assay

The treated cells were washed twice with cold PBS and lysed with RIPA lysis buffer (Beyotime Institute of Biotechnology, Nantong, China). The cells lysates were quantified with the BCA Protein Assay Kit (Thermo, Rockford, USA) according to the manufacturer's instructions. In total, 50 *μ*g of each sample was electrophoresed using a sodium dodecyl sulfate-polyacrylamide gel electrophoresis (SDS-PAGE) and transferred onto Hybond nitrocellulose membranes (Amersham, USA). After being washed briefly with TBS-T and blocked with 5% nonfat milk for 1 h, the membranes were washed and probed with the appropriate antibody overnight at 4°C with shaking. After washing with TBS-T, the membranes were incubated with 1 : 5,000 dilutions of the appropriate secondary antibody at room temperature for 1 h. The proteins were visualized using ECL chemiluminescence reagents (Thermo, Beijing, China) and exposed to Kodak X-ray films.

### 2.6. Statistical Analysis

Statistical analysis was carried out with the paired samples* t*-test. A value of *P* < 0.05 was considered statistically significant.

## 3. Results

### 3.1. Icaritin Inhibited Proliferation of Burkitt Lymphoma Cell Lines

**Figure 1:**
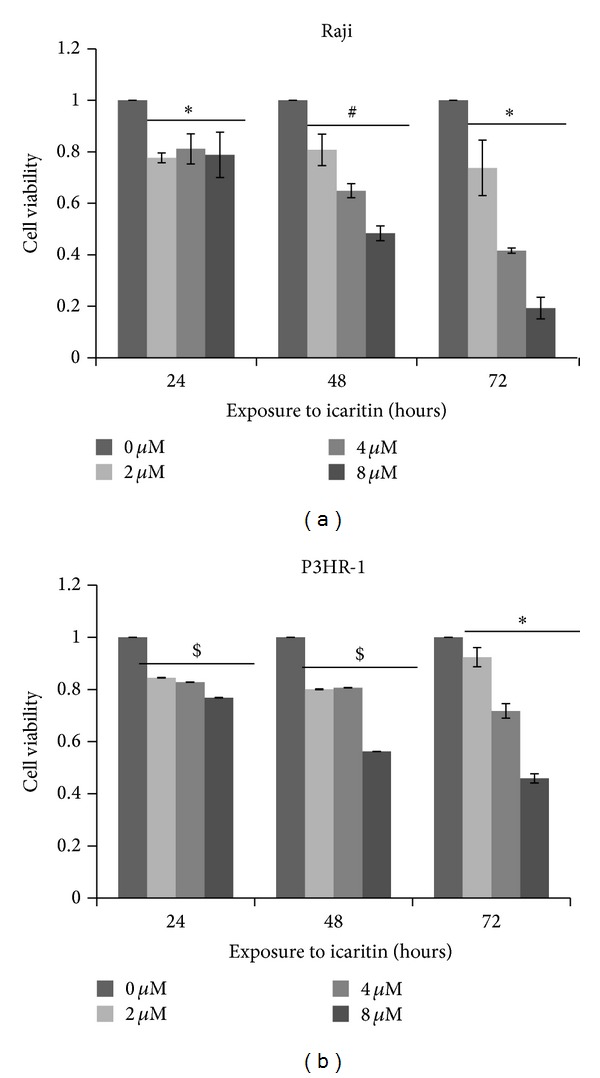
Icaritin inhibited cell growth of Burkitt lymphoma cell lines. The Raji and P3HR-1 cells were treated with DMSO (0 *μ*M icaritin) or the indicated concentrations of icaritin for the indicated time. The cells were harvested, and the cell viability was evaluated using the MTT assay. Data was expressed in mean ± SD from three independent experiments (*P < 0.05, ^#^ *P* < 0.025, and ^$^ *P* < 0.0005 compared to control cells, 0 *μ*M).

Previous studies have shown that icaritin inhibited the growth of various malignant cells [2–4, 6–9]. To determine whether icaritin inhibits the growth of Burkitt lymphoma cells, Raji and P3HR-1 cell lines were incubated with various concentrations of icaritin for 24, 48, or 72 h. The MTT assays showed that icaritin significantly inhibited the growth of both cell lines in a dose-dependent manner (Figure 1), which indicated that icaritin has antitumor activity on lymphoid malignancies such as Burkitt lymphoma. The IC50 values of icaritin on Raji cells for 24, 48, and 72 hours were 164.14 ± 112.94, 9.78 ± 1.85, and 3.6 ± 0.81 *μ*M while the IC50 values on P3HR-1 cells for indicated time were 9204.11 ± 897.75, 17.69 ± 0.03, and 9.72 ± 1.00 *μ*M. The so great IC50 values of icaritin on both cell lines for 24 h demonstrated that icaritin inhibits Burkitt lymphoma cells in a time-dependent manner as well.

### 3.2. Icaritin Induced S-Phase Arrest in Raji Cells

**Figure 2:**
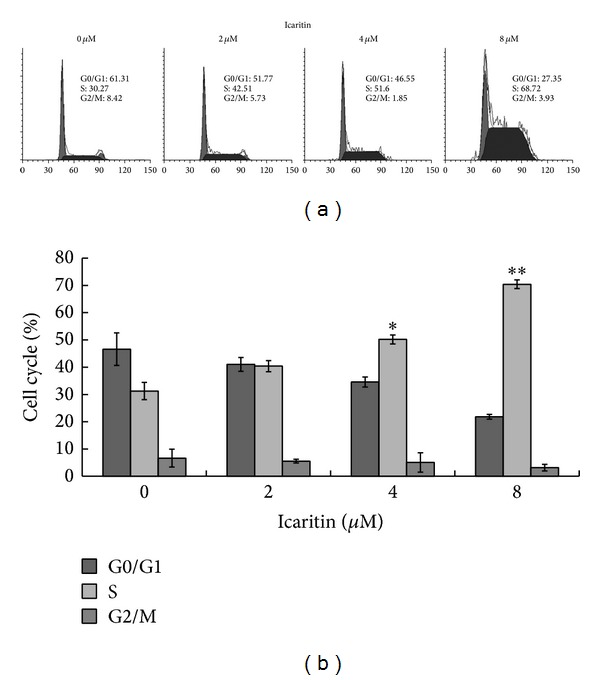
Icaritin induced S-phase arrest in Raji cells. (a) The cells were treated with DMSO or the indicated concentrations of icaritin for 48 h. The cells were evaluated using PI staining and flow cytometric analysis. The experiments were repeated, and the data from representative experiments are shown. (b) The distribution of cell cycles with the mean ± SD is shown (*P < 0.025; **P < 0.005 compared to control cells, 0 *μ*M).

Previous studies have shown that icaritin induces cell cycle arrest accompanied with decreased cell proliferation [3, 4, 9]. To investigate whether the inhibition of growth by icaritin in Burkitt lymphoma cells correlated with cell cycle arrest, we tested the cell cycle distribution of Raji cells that were treated with icaritin using flow cytometry. As shown in Figure 2, in Raji cells, icaritin increased the percentage of S-phase and reduced the population of G0/G1 phase without a concomitant increase in M phase, which indicated that icaritin mainly arrests cell cycle in S-phase, thus retarding cell growth.

### 3.3. Icaritin Induced Apoptosis in Raji Cells

**Figure 3:**
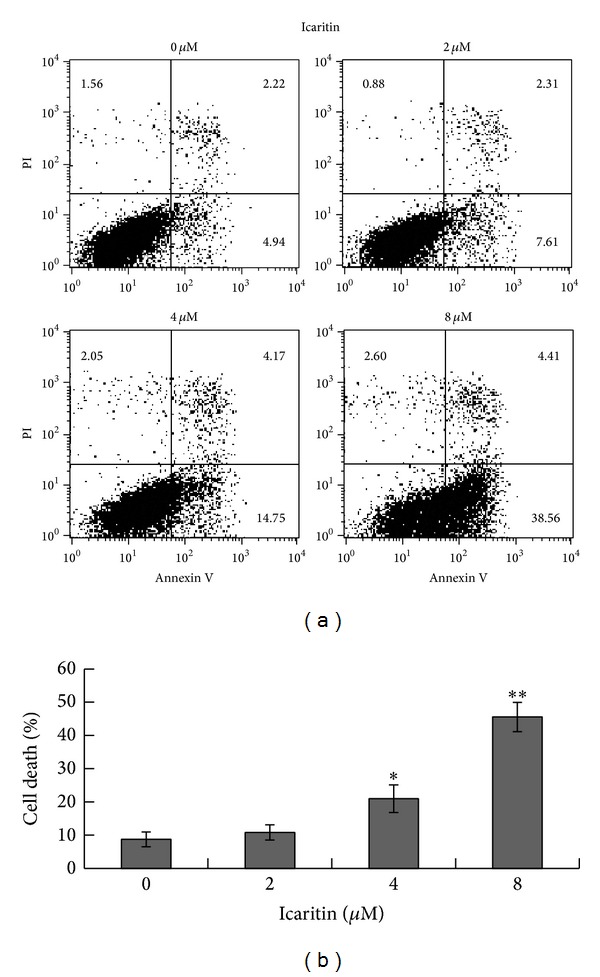
Icaritin induced cell death in Raji cells. (a) The cells were treated with DMSO or the indicated concentrations of icaritin for 48 h. The cells were analyzed with flow cytometric analysis after Annexin V and PI staining. The experiments were repeated, and the data from representative experiments are shown. (b) Total percentages of dead cells with the mean ± SD are shown (*P < 0.05; **P < 0.001 compared to control cells, 0 *μ*M).

**Figure 4:**
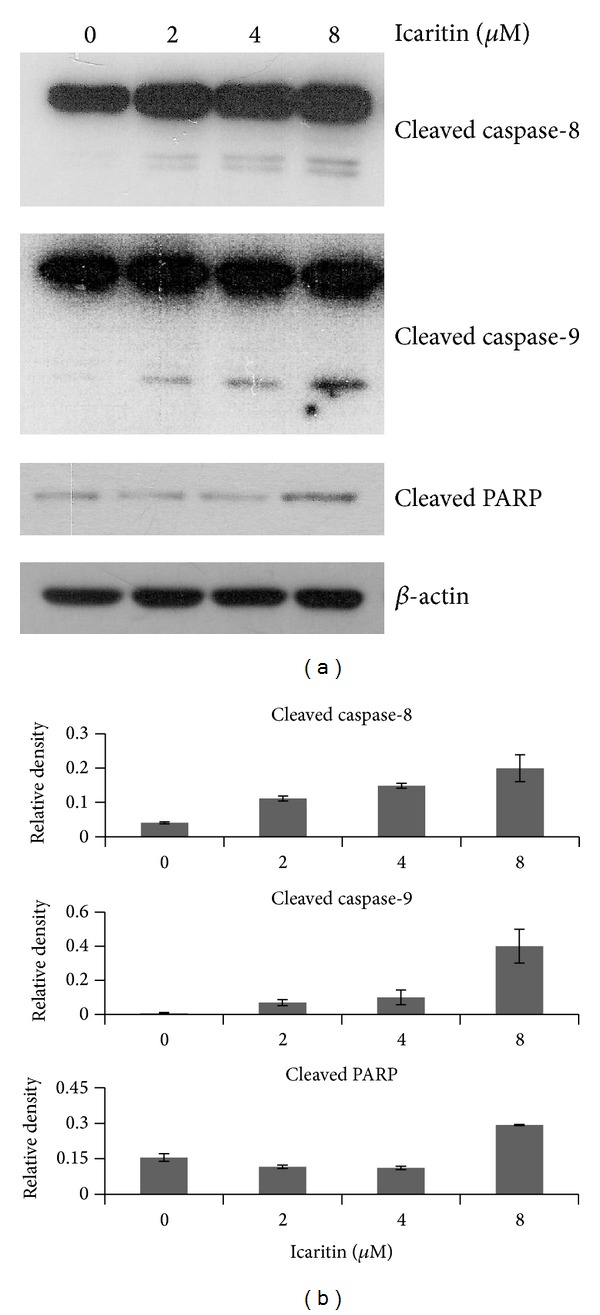
Icaritin activated apoptotic pathways in Raji cells. (a) Icaritin treatment d induced the cleavage of caspase-8, caspase-9, and PARP. The cells were treated with DMSO or different concentrations of icaritin for 48 h. The cell lysates were subjected to western blot analysis with antibodies against cleaved PARP, caspase-8, and caspase-9. *β*-actin was used as a loading control. The experiments were repeated and the data from representative experiments are shown. (b) Expression level of each protein was estimated by densitometry and presented as a ratio to the loading control *β*-actin.

In various present studies, icaritin induces cell apoptosis in many solid tumors [2–7, 10, 11] and myeloid original malignancies [8, 9]. To determine whether icaritin induces apoptosis in lymphoid malignant cells, Raji cells were treated with different concentrations of icaritin for 48 h and Annexin V-FITC and PI fluorescence assays were performed to evaluate necrotic cells as well as early stage and late stage apoptotic cells. The results showed a significant accumulation of the early and late stage apoptotic cells in icaritin treated Raji cells with dose-dependent manner (Figure 3). We then investigated whether the apoptosis induced by icaritin is associated with proteolytic activation of caspase-8 and caspase-9, two of the classic markers of mitochondrial apoptotic pathway. As shown in Figure 4, icaritin resulted in a significant accumulation of cleaved caspase-8 and cleaved caspase-9. The activation of caspases and cell apoptosis were further confirmed by assessing the cleavage of PARP, a substrate of activated caspases, in icaritin treated Raji cells. As shown in Figure 4, the 89 KDa cleaved fragment of PARP was identified. These results indicated that icaritin induced apoptosis in this lymphoid malignant cell line may be related to mitochondrial apoptotic pathway activation.

### 3.4. Icaritin Downregulated Expression of c-Myc and Bcl-2 in Raji Cells

**Figure 5:**
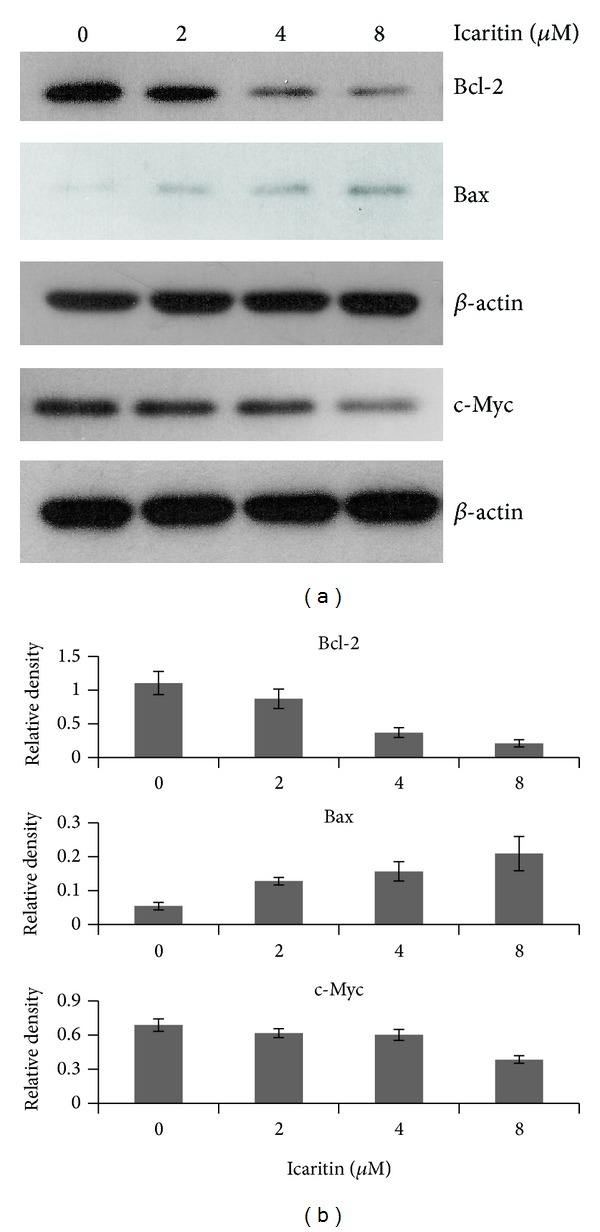
Icaritin reduced c-Myc and Bcl-2 while it increased Bax in Raji cells. (a) The cells were treated with DMSO or different concentrations of icaritin for 48 h, and the cell lysates were subjected to western blot analysis with antibody against c-Myc, Bcl-2, and Bax. *β*-actin was used as a loading control. The experiments were repeated and the data from representative experiments are shown. (b) Expression level of each protein was estimated by densitometry and presented as a ratio to the loading control *β*-actin.

Burkitt lymphoma is a so-called double-hit lymphoma that is defined by chromosomal breakpoint affecting the MYC/8q24 locus in combination with another recurrent transposition, mainly a t(14;18)(q32;q21) involving BCL2 [12]. The overexpression of MYC and BCL2 leads to enhanced proliferation [13] and reduced apoptosis [12] in transformed B cells. Moreover, the latest papers demonstrated the inhibition of c-Myc [8] and Bcl-2 [9] caused by icaritin in both acute and chronic myeloid leukemia. To probe the mechanism underlying icaritin-inhibiting growth of Burkitt lymphoma cells, the expression of c-Myc, Bcl-2, and Bax was examined by western blot. As shown in Figure 5, icaritin treatment resulted in the significant decrease of c-Myc and Bcl-2 protein and the increase of Bax protein in a dose-dependent way. These data indicated that icaritin was able to inhibit cell proliferation and induce apoptosis by influencing the two critical factors, c-myc and bcl-2, in Raji cells.

## 4. Discussion

In this study, we observed the antitumor effect of icaritin in Burkitt lymphoma cell lines, Raji and P3HR-1. Furthermore, we investigated the potential mechanisms in Raji cells. Our results showed that icaritin significantly inhibits the proliferation of the two Burkitt lymphoma cell lines in a dose- and time-dependent manner (Figure 1). Then we used Raji cells as a model and found that icaritin causes the accumulation of S-phase cells (Figure 2) and induces cell apoptosis (Figure 3) with activation of caspase-9 and caspase-8 (Figure 4). Finally, we demonstrate that icaritin decreases the two critical signal factors, c-Myc and Bcl-2 (Figure 5), which promote cell proliferation and survival in Burkitt lymphoma cell [12].

Previous studies have shown that icaritin possesses extensive antitumor activities on human solid and hematologic tumor cells including endometrial cancer Hec1A cells [2], prostate cancer PC-3 cells [3], breast cancer MCF-7 and MDA-MB-453 cells [4], hepatoma HepG2 cells [6], renal carcinoma 786-0 cells [11], chronic and acute myeloid leukemia primary cells, and cell lines [8, 9]. However, the report about antitumor activities of icaritin in lymphoid malignancies is still not available. Herein, we, for the first time, demonstrated that icaritin has the potential against Burkitt lymphoma by inhibiting cell proliferation and inducing cell apoptosis.

It is well established that cell cycle is linked to cell proliferation and apoptosis [14, 15]. As a novel anticancer agent, icaritin induces cell death that is accompanied by cell cycle arrest in different phases in various malignancies: mammary cancer MCF-7 cells in G2/M phase [4], prostate cancer PC-3 cells in G1 phase [3], chronic myeloid leukemia K562 cells in G1 phase [9], and acute myeloid leukemia cell lines in S-phase [8]. In this study, we found that icaritin arrests Raji cells in S-phase in a dose-dependent manner (Figure 2). Apparently, the effect of S-phase arrest of icaritin is contributed to the growth inhibition of Raji cells caused by it. As to why icaritin results in different cell phase arrest in various malignant cell lines with the similar inhibition of cell growth, this may be related to the various cell internal environments in different malignant cells.

Notably, icaritin may potently trigger apoptosis in Raji cells that had been treated for 48 h. The phenomenon that icaritin just caused a significant accumulation of Annexin V positive but PI negative cells at 48 h, which indicated that the cell membrane is still undamaged (Figure 3), could elucidate the absence of sub-G1 phase cells at 48 h (Figure 2). To investigate the way by which icaritin prompts apoptosis in Raji cells, we detected and found that icaritin induces the activation of caspase-8, caspase-9 and the cleavage of PARP, which are similar to the report of Tong et al. [2] and Huang et al. [3]. It has been documented that mitochondria play a pivotal role in the signal transduction of apoptosis [16]. The activation of caspase-9 and caspase-3 and subsequent cleavage of PARP and release of cytochrome c from mitochondria imply the activation of mitochondrial-mediated caspases cascade pathway [17]. So, the activation of these proteins that we detected in the pathway suggests that icaritin induced apoptosis in Raji cells might be related to mitochondrial-mediated caspases pathway.

As one of the* double hit* lymphoma, Burkitt lymphoma cells obtain two critical uncontrolled genes: BCL2 and MYC [12], which make the malignant cells survive and proliferate out of control. Therefore, agents that target one or both of the two factors are able to induce apoptosis and are considered to be the potential drugs that can be used to treat Burkitt lymphoma [12, 18, 19]. Moreover, icaritin shows the ability to decrease the level of Bcl-2 and c-Myc proteins in several studies [2, 4, 8, 9]. To understand the influence of icaritin on the two crucial factors in Burkitt lymphoma cells, we detected them with western blot and found that both of them were downregulated by icaritin in dose-dependent manner (Figure 5). Thus, though the more imperative exploration is needed, these data that we obtained have suggested that Bcl-2 and c-Myc were involved in the inhibition of proliferation and survival caused by icaritin, especially in the DH lymphomas.

## 5. Conclusions

In conclusion, to our knowledge, we have reported for the first time that icaritin shows antitumor effect in lymphoid malignant cell lines. Our experimental results have shown that icaritin is able to inhibit cell growth and induce apoptosis in Burkitt lymphoma cell lines. The underlying mechanisms of icaritin antilymphoma may be related to inhibition of bcl-2 and c-myc. However, considering that the pan influence of icaritin on MAPK/ERK/JNK and JAD2/STAT3/AKT signals has been reported in various tumors, further researches in more lymphoid malignancies and more in-depth experiments remain needed.
